# Lived Experience is Our Superpower: Narratives from Four Global Shapers, World Economic Forum (WEF) Youth Mental Health Leaders Journeys

**DOI:** 10.1371/journal.pmen.0000243

**Published:** 2025-08-12

**Authors:** Matthew Jackman, Matin Moradkhan, Sanjana Gupta, Himadri Thapliyal

**Affiliations:** 1 University of Sydney, Camperdown, Australia; 2 University of British Columbia, University Blvd, Vancouver, British Columbia, Canada; 3 Gujarat University, Navrangpura, Ahmedabad, Gujarat, India; 4 National Forensic Sciences University Gandhinagar, Gandhinagar, India; PLOS: Public Library of Science, UNITED KINGDOM OF GREAT BRITAIN AND NORTHERN IRELAND

## Lived experience and leadership

Leadership is not just about authority or position,it can be about using one’s journey to inspire, empower, and create meaningful change. Each of us faces challenges that shape who we are, but it is how we respond to struggles which has the potential to define our resistance and leadership approaches. Through resilience, empathy, and a deep sense of purpose, we can turn our most difficult moments into powerful tools for personal and collective transformation.

This opinion aims to exemplify how individuals have embraced their lived experiences,ranging from grief, trauma, and distress, driving our leadership journeys. From navigating cultural shocks to overcoming personal loss, the stories of Matin, Sanjana, Himadri, and Matthew illustrate the immense potential of self-determination and transformation from pain into purpose. They show how adversity, when reframed and understood, can become a catalyst for advocacy and social change. Through our experiences, we demonstrate that leadership is about empathic, impactful, and purpose-driven actions. These stories through first person accounts highlight how adversity can become a source of strength, not just for personal growth but for inspiring others and creating a more compassionate world.

We hope that all individuals, when appropriate and when they feel safe enough to do so, can use their lived experiences as a source of leadership, resistance and/or activism, and empowerment for social change. We call for resisters, disruptors, activists and/or leaders toembrace their mental health challenges to authentically transform society. Madness can be a gift, when harnessed.

## How to use your experiences to shape your leadership approach: Matin’s lived experience

Each of us has a unique story of struggle, but it’s how we transform that struggle into purpose that empowers us to create change. My journey, marked by experiencing isolation, anxiety, and grief, has shown me that our most challenging experiences can ignite our inner strength and drive us toward self-determination.

As a Canadian-Iranian immigrant, I faced cultural shock, academic pressure, and language barriers when I moved to Canada to pursue my studies at the University of British Columbia. Initially, these challenges led me to a place of deep anxiety and feeling hopeless. I considered dropping out, feeling overwhelmed by the weight of my unmet expectations. However, I refused to let fear define me. Reaching out for help, I was inspired by the encouragement of a fellow student, and I pushed forward, determined to face my struggles head-on [1].

In 2020, as the world battled the pandemic, I faced the added trauma of losing a mentor followed by losing a family member to COVID-19 in Iran. The guilt of surviving while my family suffered was devastating. Yet, this grief became the catalyst for a powerful transformation. Rather than giving up, and staying helpless, I channeled my pain into a passionate drive for change [2]. I immersed myself in global health studies, volunteering to shape my knowledge and gaining a sense of agency in advocating for policy changes, particularly in mental health. My experiences created a deep sense of purpose and leadership, and that determination has gotten stronger after getting elected as the vice-curator of the Vancouver hub of the Global Shapers Community [3].

Through these experiences, I learned that agency is not about avoiding challenges but about finding strength in the face of adversity. Our lived experiences, especially those filled with pain and hardship, can empower us to rise, reclaim control, and use our voices to create positive change in the world. This newfound self-determination has become my superpower—helping me lead with empathy and purpose and enabling me to advocate for those who, like me, once felt powerless.

## Change is about fighting and persistence: Sanjana’s and Himadri’s lived experience

### Sanjana: From trauma to self-determination

Lived experience is the ultimate teacher of agency, and my journey through grief, trauma, and mental health struggles has taught me that even the darkest moments can lead to profound self-discovery and empowerment. Much can be gained if an individual recognizes that their experience is a source of valuable expertise [4]. Losing my father at a young age shattered the foundation of my life. This tragic loss left me with a deep sense of insecurity and mistrust, making it hard to find a sense of belonging. For years, I struggled with emotional pain, and I couldn’t understand why I felt so lost.

It wasn’t until much later that I realized my experiences had been shaping me into someone who could navigate life with greater resilience. In 2021, after a traumatic accident, I faced panic attacks, depression, and overwhelming anxiety. But, rather than allowing these challenges to define me, I recognized them as signals urging me to reclaim my agency. Therapy, though initially difficult to commit to, eventually became a turning point. Establishing a collaborative, mutual partnership to work together to set goals and complete treatment tasks was key [5]. Finding a therapist who resonated with me allowed me to begin the work of healing and self-empowerment.

In embracing my journey, I learned that agency lies in our ability to trust ourselves, rebuild from brokenness, and take control of our own healing. I began to see the power of my own choices—the decision to seek help, the willingness to embrace hope, and the strength to rebuild my relationships. Now, as a Shaper, I use my lived experience to advocate for mental well-being through projects like *Mind Matters* [6]. My goal is to empower others to find their own agency, to give them the hope and strength to rise from their own struggles, and to create communities where people are supported and encouraged to thrive.

My lived experience has taught me that true self-determination comes from within. It’s about choosing to continue despite the odds and believing that our journey, no matter how painful, has the power to shape us into leaders who inspire others to do the same.

### Himadri: From death to self-determination

Life often presents us with experiences that test our will to survive, but through these trials, we discover the immense power of our own agency. My life took a drastic turn when, during the second wave of COVID-19, both my parents contracted the virus. In the midst of this, I too fell critically ill. The fear of losing my life made me question everything—what is the meaning of life? What does it mean to truly live?

I was locked in a room, struggling to breathe, my oxygen levels dangerously low, and my body wasting away. As I lay on the bathroom floor, my pet cat by my side, I thought about how fragile life is. Despite my condition, I found the courage to drive myself to get an MRI, which revealed that 75% of my lungs were damaged. The trauma of being admitted to a COVID-19 containment facility, surrounded by those losing their loved ones, was a stark reminder of life’s uncertainty [7]. But in this moment of terror, I also discovered an inner strength I never knew existed.

Surviving that experience was a turning point. It taught me that health and life are not guaranteed, and we must take full ownership of our well-being. After recovering, I began to shift my focus to what truly matters: relationships, health, and emotional resilience. These values became the foundation for my newfound sense of agency and self-determination [8]. A later life-altering experience, giving birth prematurely due to a rare tumor, reinforced this realization. The strength I drew from these traumatic events became my superpower—the ability to face life’s most challenging moments with determination and courage [9].

Through my experiences with death, illness, and recovery, I have learned that agency is the ability to choose to keep going, even when faced with the most difficult circumstances. It is the power to determine what matters, to take ownership of one’s life and health, and to recognize the strength within to keep moving forward. These lessons have given me a deep sense of self-determination and an unwavering commitment to living fully.

### The power of reframing: Matthew Jackman’s lived experience of transforming responses to trauma into International Mad activism

Sometimes, the most difficult life experiences can become the foundation for personal agency and activism. I grew up in a family riddled with intergenerational grief and loss, complex trauma, and distress and by the age of nine, I experienced the devastating loss of my mother to suicide. This loss, coupled with the trauma of living in foster care, facing emotional neglect, and coming to terms with my sexuality in a homophobic low socio-economic regional upbringing, would shape my sense of self for many years [10].

At a young age, I took on the role of caregiver for my younger siblings, navigating life with a single father struggling financially, and relying on financially stable partners who were abusive and neglectful. I battled my own mental health challenges, eventually being diagnosed by Psychiatry with Bipolar Affective Disorder, which I understand through a non pathological lens of trauma and responses to grief, loss, disconnection, injustice and rage. I spent years navigating the psychiatric system, facing stigma, sanism, discrimination and the challenges of medication side effects and psychiatric oppression. But instead of letting these experiences defeat me, they ignited a deep desire for change. I realized that I could use my lived experience to fuel my Mad activism and advocate for those who had suffered as I had [11].

Through my struggles with mental distress, family trauma, and systemic issues, I developed a profound sense of agency. I understood that true activism comes from recognizing the root causes of distress and fighting for a more just and inclusive system. My experiences as a queer, non-binary individual who faced homophobia and at times transphobia despite often cis passing, fuelled my determination to create systemic change [12]. I now use my lived experience as a superpower at times, despite its very real impairing and psychic pain aspects, to channell my distress into a platform for advocacy.

My journey has taught me that agency is about transforming trauma into action. By using our voices, our stories, and our experiences, we can both heal and advocate for those whose struggles have been ignored or marginalized through our own narrative. My lived experience has empowered me to become an activist for systemic change, working toward a world where mental health is understood holistically, where people’s identities are affirmed, and where everyone can thrive [13]. Far from being solely destructive, these altered and extreme states of Madness offered me spiritual awakening and revelation, which offered mea unique perspective on the world. Through Mad Studies, I’ve come to view 'mania' as a complex valuable source of mad wisdom and lived expertise, similarly for experiences of 'depression', thoughts of suicide and actions to end my life, which have challenged traditional psychiatric frameworks and embracied its potential for strength, insights and creativity [14].

## Lived experience is expertise and a source of transformative change

Our collective experiences underscore a powerful truth: our lived experiences, especially those marked by hardship and adversity, can become the foundation for transformative leadership. By embracing pain, grief, and struggle, we not only cultivate resilience but also unlock a deeper sense of purpose and agency that empowers us to create meaningful change. These experiences teach us that leadership is not defined by our rolesand circumstances, but by our ability to turn our personal journeys into sources of strength, compassion, and advocacy. As Matin, Sanjana, Himadri, and Matthew’s stories illustrate, it is through our struggles that we can sometimes discover our true potential to lead, inspire, and uplift others by proving that the most profound impact often comes from those who have lived through the toughest challenges and emerged stronger, wiser, and ready to shape a better future. Lived experience is expertise, it is a discipline, a social movement and a valued knowledge in activating for values based systems change towards rights and justice based leadership and activism.
